# Association between postoperative nadir platelet count and postoperative cardiovascular complications following septal myectomy in patients with hypertrophic cardiomyopathy: a retrospective cohort study

**DOI:** 10.1186/s12872-024-03724-2

**Published:** 2024-01-18

**Authors:** Qianqian Fan, Zhihong Lu, Yonghui Wang, Lini Wang, Hui Zhang, Ziyu Zheng, Hailong Dong, Lize Xiong, Chong Lei

**Affiliations:** 1 Department of Anesthesiology and Perioperative Medicine, Xijing Hospital, Fourth Military Medical University, 127 West Changle Road, Xi’an, Shaanxi 710032 China; 2 Department of Anesthesiology and Translational Research Institute of Brain and Brain-like Intelligence, Shanghai Fourth People’s Hospital Affiliated to Tongji University School of Medicine, Shanghai, 200081 China

**Keywords:** Cardiovascular complication, Hypertrophic cardiomyopathy, Nadir platelet count, Septal myectomy

## Abstract

**Background:**

Platelet count is associated with cardiovascular risk and mortality in several cardiovascular diseases, but the association of the nadir platelet counts post-septal myectomy with the cardiovascular complication risk in hypertrophic obstructive cardiomyopathy patients remains unclear.

**Methods:**

This retrospective cohort study reviewed all adult patients who underwent septal myectomy at a single tertiary referral center over a 5-year period. Postoperative nadir platelet count was defined as the lowest platelet count in the first 4 postoperative days or until hospital discharge. The composite outcome included cardiovascular death, myocardial infarction, heart failure, malignant arrhythmia, cardiac tamponade, and major bleeding events within 30 days postoperatively. Univariable and multivariable logistic regression and restricted cubic spline models were used to assess the association between postoperative nadir platelet count and the 30-day postoperative cardiovascular complication risk.

**Results:**

Among the 113 enrolled patients, 23 (20.4%) developed cardiovascular events within 30 days postoperatively. The incidence of postoperative cardiovascular complications was significantly higher in patients with a nadir platelet count ≤ 99 × 10^9^/L than in those with a nadir platelet count > 99 × 10^9^/L (33.3% vs. 7.1%, crude risk ratio: 4.67, 95% confidence interval: 1.69–12.85, *P* < 0.001). Multivariable logistic regression revealed that postoperative nadir platelet count was negatively associated with 30-day postoperative cardiovascular complications (adjusted odds ratio: 0.97; 95% confidence interval: 0.95–0.99; *P* = 0.005) and the association was linear (*P*_nonlinearity_ = 0.058) after full adjustment. The association between nadir platelet count and cardiovascular complications within 30 days post-surgery was consistent in all predefined subgroups (P_interaction_ > 0.05).

**Conclusion:**

The postoperative nadir platelet count was significantly associated with the 30-day post-myectomy risk of cardiovascular complications in hypertrophic obstructive cardiomyopathy patients.

**Trial registration:**

This trial was registered at ClinicalTrials.gov (NCT04275544).

## Introduction

Transaortic septal myectomy is the gold standard and primary treatment option for hypertrophic obstructive cardiomyopathy (HOCM) patients with significant left ventricular outflow tract (LVOT) obstruction and drug-refractory heart failure symptoms [1]. The annual rate of septal myectomy is approximately 1.5 procedures per million people [2]. Approximately 25% of septal myectomy patients experience postprocedural cardiovascular events, including mortality in 5.9% of cases [3, 4]. The etiology of cardiovascular events after septal myectomy is complex, and several factors, such as aging, ischemia–reperfusion, cardiopulmonary bypass, and inflammatory responses may be involved [2, 3, 5, 6].

Platelet count measurement, a clinical routine for perioperative management, is linked to clinical outcomes in surgical and non-surgical patients [7–11]. Decreased platelet count and platelet dysfunction have been associated with heart failure [9, 10], and myocardial infarction [11] in non-surgical patients. Thrombocytopenia was also found to be correlated with hemostatic dysfunction [12], atrial fibrillation [13], and increased stroke, acute kidney injury, and mortality [14] in cardiac procedures. The mechanism underlying these relationships may extend beyond platelet roles in hemostasis and thrombosis. Platelets appear to be crucial modulators of inflammation and immune responses [15]. They promote immune cell activation and recruitment to endothelial injury sites by regulating cytokines and endogenous biological mediators, which may inhibit inflammation and help to kill pathogens [15]. Thus, thrombocytopenia could hinder the body’s ability to combat immune responses and control inflammation. Platelet dysfunction is also closely linked to cardiovascular complication risk markers in patients with hypertrophic cardiomyopathy, such as LVOT obstruction and left ventricular hypertrophy [16, 17]. Nevertheless, the association between postoperative nadir platelet count and cardiovascular complications after septal myectomy in HOCM patients is unknown.

This study investigated whether a lower postoperative nadir platelet count is associated with increased 30-day postoperative cardiovascular complications in HOCM patients after septal myectomy.

## Methods

### Study design and setting

Ethical approval for this single-center, retrospective cohort study was obtained from the Institutional Review Board of Xijing Hospital (No. KY20200121-C-1). The need for written informed consent was waived by the Xijing Hospital ethics committee due to the retrospective nature of the study. This trial was registered on 19/02/2020 at ClinicalTrials.gov (NCT04275544). Strengthening the Reporting of Observational Studies in Epidemiology guidelines was followed in this study.

### Subjects and data obtainment

Adult HOCM patients (aged ≥ 18 years) who underwent transaortic septal myectomy at Xijing Hospital between October 2013 and December 2018 were eligible. Patients who underwent emergency surgery or had presurgical hematological disorders were excluded.

Patient demographic and perioperative data were independently extracted from the hospital’s electronic healthcare records (EHRs) by two trained investigators who were blinded to the study purpose. The patients’ 30-day postoperative prognosis was obtained through interviews and telephone follow-up or by reviewing EHRs if the postoperative hospital stay exceeded 30 days. The primary investigator resolved logical errors, core data omission, and investigator discrepancies during data collection.

### Exposure

The exposure variable was postoperative nadir platelet count, which was defined as the lowest platelet count during the first 4 days postoperatively or until hospital discharge, whichever occurred first [18, 19]. Platelet count data, obtained from the EHRs, were measured using a Sysmex XN-3000 hematology analyzer (Sysmex Corporation, Kobe, Japan) at the hospital’s central laboratory. The platelet count reference range was 125–350 × 10^9^/L.

### Outcomes

The study outcome was the 30-day postoperative cardiovascular complications, a composite of cardiovascular death, myocardial infarction [20], heart failure [21], malignant arrhythmia (including emerging atrial fibrillation or flutter, ventricular tachycardia, ventricular fibrillation, and conduction disturbances requiring permanent pacing), cardiac tamponade, and major bleeding events [22]. Doctors’ diagnoses in the EHRs, and investigators’ manual review of the medical records were used to evaluate cardiovascular complication occurrence. Patients were deemed to have had a cardiovascular complication if they met the criteria during the medical review, even if not documented in the EHRs. The exact time at which these cardiovascular complications occurred was recorded in detail. Complications that occurred before the nadir platelet count were omitted from the composite endpoint analysis.

### Covariates

Covariates considered in the analysis included patient demographics, comorbidities, medication history, laboratory values, preoperative echocardiographic parameters, intraoperative surgical variables, and postoperative blood management within 4 days postoperatively.

Demographic data included age, sex, body mass index, syncope history, and family history of sudden cardiac death and hypertrophic cardiomyopathy.

Clinical characteristics included the duration of clinical symptoms, pre-existing comorbidities (hypertension, coronary artery disease, diabetes, pulmonary arterial hypertension, asthma, stroke, and atrial fibrillation), and medication history.

Laboratory values included baseline coagulation test results, hemoglobin levels, preoperative platelet count, and postoperative nadir platelet count. Per the institutional protocol, preoperative complete blood counts and blood coagulation values were measured within 7 days preoperatively. Postoperative complete blood counts were measured at least once daily during the patients’ stay in the intensive care unit and then once every other day after transfer to the ward.

Preoperative transthoracic echocardiographic parameters included the left atrial diameter, ejection fraction, maximal interventricular septal thickness, systolic anterior motion of the mitral valve, and resting LVOT gradient.

We also recorded information on intraoperative surgical variables (anesthesia time, surgery time, cardiopulmonary bypass time, with or without a concomitant procedure, intraoperative blood transfusions and fluid management, and duration of hypotension during surgery). Hypotension was diagnosed when the mean arterial pressure decreased by 20% from the baseline or when the systolic blood pressure was below 90 mmHg. The duration of intraoperative hypotension was defined as the time (in minutes) of hypotension recorded in the electronic anesthesia system during surgery.

### Statistical analysis

Based on a prior review of 1-year EHRs, we deduced that the incidence of 30-day postoperative cardiovascular complications was 10.0% in HOCM patients with higher nadir platelet counts. We calculated that a sample size of 106 subjects would provide 80% power to detect a relative risk of 3.5 for the incidence of the primary outcomes between patients with lower and higher nadir platelet counts (categorized by the median value) at a two-sided alpha level of 0.05 and a 5% dropout rate.

Continuous variables are described as the mean (standard deviation) or median (25th − 75th percentiles), as appropriate. The normality of data distribution was examined using the Shapiro–Wilk test. Categorical variables are described as counts (percentages) and were compared using chi-squared or Fisher’s exact tests (for expected counts < 5).

Univariable and multivariable logistic regression models were used to assess the relationship between the postoperative nadir platelet count and cardiovascular events in the study population. Covariates were chosen if one of the following criteria were satisfied: (1) literature-based confounders, (2) covariates with *P* < 0.05 in univariable analyses, and (3) effect size of the exposure changed > 10% after including or omitting the variable as a covariate in the model.

A restricted cubic spline with four knots (at the 5th, 35th, 65th, and 95th percentiles of the nadir platelet count) was used for flexible modeling of the association between changes in nadir platelet count and cardiovascular complications. We evaluated three to five knots but opted to use four knots, as this minimized the Akaike information criterion. The spline model was then full adjustment.

We also performed subgroup analyses using logistic regression models to investigate whether the relationship between the postoperative nadir platelet count and outcome events varied by sex (male vs. female), age ( ≤ 60 vs. > 60 years), baseline platelet count (> median vs. ≤ median value), and plasma transfusion volume at 4 days postoperatively (> median vs. ≤ median value). The significance of interaction effects was assessed in each subgroup.

The final analysis did not include patients with missing postoperative platelet records, missing 30-day postoperative cardiovascular complication information, or > 10% missing covariate data. If < 10% covariate data were missing, missing values were estimated using informative or mean imputation, as appropriate. Two-tailed *P* values < 0.05 indicated statistical significance. Data analyses were conducted using IBM SPSS Statistics for Windows (version 24.0; IBM Corporation, Armonk, NY, USA) and the Free Statistics analysis platform.

## Results

### Baseline characteristics and perioperative variables of this cohort

**Fig. 1:**
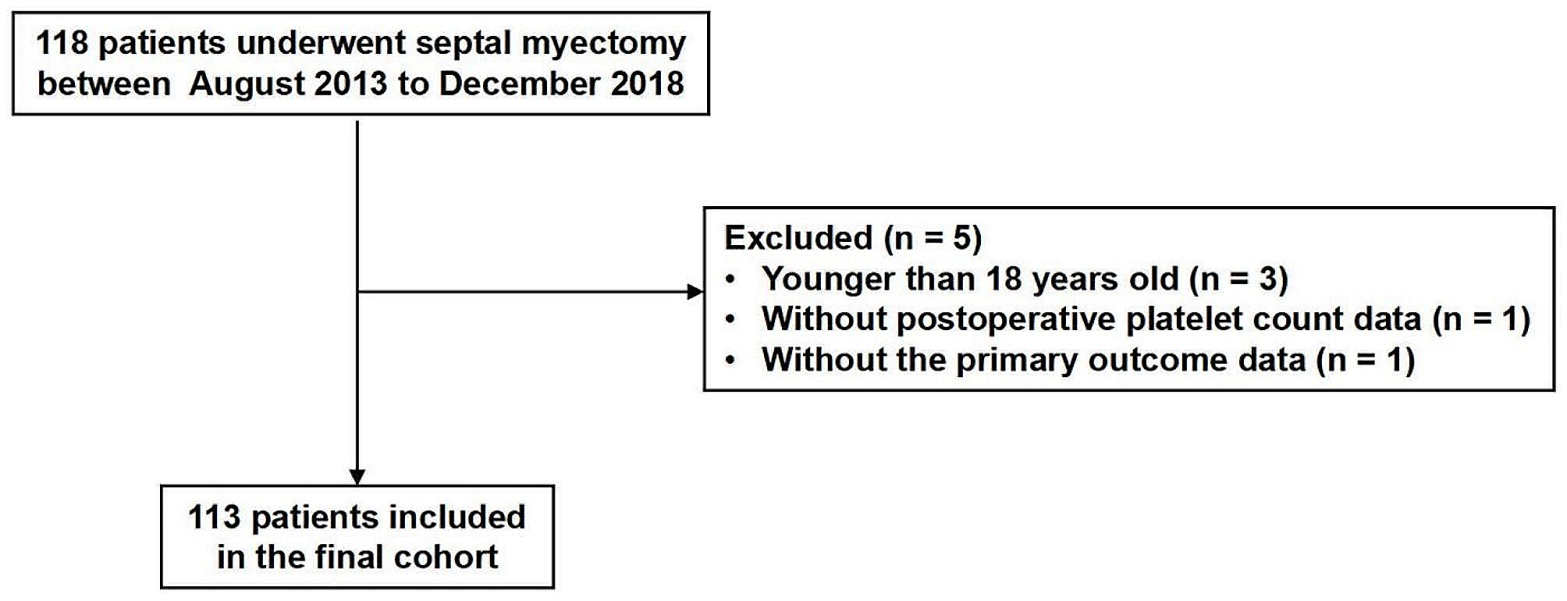
Study flowchart

In total, 118 unrelated patients met the study eligibility criteria. After excluding five patients, 113 subjects were included in the final analysis (Fig. 1). The baseline characteristics and perioperative variables of the patients are presented in Table 1. The mean age of this cohort was 47.6 ± 12.8 years old and 48 (42.5%) patients were female. The median value of the postoperative nadir platelet count was 99.0 (80.0, 137.0) × 10^9^/L.

**Table 1:** Baseline characteristics and perioperative variables of the study population

Variable	Total (*n* = 113)	Platelet Nadir > 99 × 10^9^/L (*n* = 56)	Platelet Nadir ≤ 99 × 10^9^/L (*n* = 57)	*P*-value
Age (years)	47.6 ± 12.8	46.3 ± 11.6	48.9 ± 13.8	0.284
Female	48 (42.5)	22 (39.3)	26 (45.6)	0.496
Body mass index (kg/m^2^)	24.3 ± 3.8	24.8 ± 4.3	23.7 ± 3.3	0.129
Family history of hypertrophic cardiomyopathy	14 (12.4)	4 (7.1)	10 (17.5)	0.093
Family history of sudden death	10 (8.8)	4 (7.1)	6 (10.5)	0.742
History of syncope	35 (31.0)	15 (26.8)	20 (35.1)	0.340
Duration of clinical symptoms (years)	3.0 (1.0, 7.0)	2.0 (1.0, 5.0)	5.0 (2.0, 10.0)	0.031
**Comorbidities**				
Hypertension	48 (42.5)	25 (44.6)	23 (40.4)	0.644
Coronary artery disease	19 (16.8)	10 (17.9)	9 (15.8)	0.769
Pulmonary arterial hypertension	11 (9.7)	4 (7.1)	7 (12.3)	0.357
Diabetes	3 (2.7)	3 (5.4)	0 (0)	0.118
Stoke	4 (3.5)	2 (3.6)	2 (3.5)	1.000
Atrial fibrillation	5 (4.4)	3 (5.4)	2 (3.5)	0.679
**Preoperative medications**				
Aspirin or Clopidogrel	15 (13.3)	7 (12.5)	8 (14)	0.81
Diuretics	6 (5.3)	3 (5.4)	3 (5.3)	1.000
β-blocker	40 (35.4)	17 (30.4)	23 (40.4)	0.267
Statin	3 (2.7)	2 (3.6)	1 (1.8)	1.000
ACEI or ARB	21 (18.6)	11 (19.6)	10 (17.5)	0.774
**Laboratory variables**				
Baseline partial thromboplastin time (s)	11.2 ± 1.5	11.2 ± 1.0	11.1 ± 1.8	0.785
Baseline activated partial thromboplastin time (s)	27.4 ± 5.2	27.2 ± 5.4	27.6 ± 5.1	0.720
Baseline international normalized ratio	1.0 (1.0, 1.1)	1.0 (0.9, 1.0)	1.0 (1.0, 1.1)	0.115
Baseline hemoglobin (g/L)	138.7 ± 22.2	136.1 ± 25.4	141.2 ± 18.4	0.224
Baseline platelet count (10^9^/L)	189.4 ± 63.3	219.6 ± 65.4	159.6 ± 44.7	< 0.001
Nadir platelet count within postoperative four days (10^9^/L)	99.0 (80.0, 137.0)	137.0 (116.5, 165.0)	80.0 (67.0, 87.0)	< 0.001
The absolute decrease from baseline in platelets (10^9^/L)	71.5 (43.8, 110.2)	66.0(29.8,94.5)	81.0 (53.0, 115.0)	0.059
Percentage decrease from baseline in platelets (%)	42.6 (29.0, 52.4)	33.8(17.5,40.0)	50.3 (42.8, 57.3)	< 0.001
**Preoperative echocardiography**				
Left atrium dimension (mm)	45.8 ± 6.4	45.6 ± 6.3	46.1 ± 6.5	0.68
Ejection fraction (%)	61.9 ± 6.0	60.9 ± 5.9	62.8 ± 5.9	0.087
Maximal septal thickness (mm)	24.2 ± 6.3	23.8 ± 5.3	24.7 ± 7.1	0.431
Resting LVOT gradient (mm Hg)	81.0 (44.0, 108.0)	73.0 (35.0, 97.2)	84.0 (53.0, 117.0)	0.098
With systolic anterior motion	93 (82.3)	45 (80.4)	48 (84.2)	0.592
**Intraoperative parameters**				
Anesthesia time (min)	273.1 ± 76.7	253.1 ± 55.0	292.7 ± 89.5	0.006
Surgical time (min)	240.8 ± 70.9	219.6 ± 51.2	261.6 ± 81.2	0.001
Cardiopulmonary bypass time (min)	121.0 (95.0, 153.0)	118.5 (85.8, 143.2)	128.0 (104.0, 172.0)	0.028
Cross-clamp time (min)	70.0 (51.0, 90.0)	69.5 (48.0, 88.0)	74.0 (52.0, 95.0)	0.115
Concomitant other procedure	36 (31.9)	16 (28.6)	20 (35.1)	0.457
Hypotension time (min)	150.0 (115.0, 200.0)	135.0 (113.8, 187.5)	155.0 (115.0, 210.0)	0.157
**Intraoperative fluid management**				
Fluid intake (mL)	1600.0 (1300.0, 2000.0)	1600.0 (1300.0, 1862.0)	1500.0 (1250.0, 2010.0)	0.966
Red blood cells (units)	2.0 (0.0, 2.0)	1.5 (0.0, 2.0)	2.0 (0.0, 2.0)	0.878
Plasma (mL)	0.0 (0.0, 200.0)	0.0 (0.0, 200.0)	0.0 (0.0, 200.0)	0.584
Total output (mL)	980.0 (700.0, 1450.0)	980.0 (700.0, 1500.0)	980.0 (700.0, 1400.0)	0.713
Blood loss (mL)	300.0 (300.0, 400.0)	300.0 (300.0, 400.0)	300.0 (300.0, 400.0)	0.548
Urine volume (mL)	400.0 (200.0, 600.0)	425.0 (200.0, 700.0)	400.0 (300.0, 600.0)	0.441
**Blood products within postoperative four days**				
Red blood cells (units)	0.0 (0.0, 2.5)	0.0 (0.0, 2.0)	2.0 (0.0, 4.0)	0.004
Plasma (mL)	740.0 (370.0, 1090.0)	550.0 (327.5, 970.0)	800.0 (400.0, 1510.0)	0.026
Cryoprecipitate	12 (10.6)	4 (7.1)	8 (14)	0.234
Platelet	5 (4.4)	2 (3.6)	3 (5.3)	1
Blood loss within postoperative four days (mL)	580.0 (370.0, 830.0)	475.0 (303.8, 662.5)	735.0 (475.0, 900.0)	0.001

Data are presented as the mean ± standard deviation, n (%), or median (25th–75th percentiles). *Abbreviations: ACEI* angiotensin-converting enzyme inhibitors, *ARB* angiotensin receptor blockers, *LVOT* left ventricular outflow tract

### Outcomes

Thirty-day postoperative cardiovascular events occurred in 23 (20.4%) patients. Among these patients, seven (6.2%) suffered cardiac-related mortality, five (4.4%) developed heart failure, five (4.4%) developed ventricular arrhythmia, seven (6.2%) required a permanently implanted cardiac defibrillator, one (0.9%) needed reoperation due to major bleeding, and three (2.7%) developed cardiac tamponade. The incidence of postoperative cardiovascular complications was significantly higher in patients with a nadir platelet count ≤ 99 × 10^9^/L than in those with a count > 99 × 10^9^/L (33.3% vs. 7.1%, crude risk ratio: 4.67, 95% confidence interval: 1.69–12.85, *P* < 0.001).

### Covariate screening

The results of the univariable logistic analysis are presented in Table 2. Baseline platelet count was arbitrarily selected for multivariable regression analysis as an important baseline covariate. Intraoperative hypotension time, red blood cell and platelet transfusion within 4 days postoperatively, and blood loss within 4 days postoperatively were excluded because the effect size of the exposure changed < 10% after including or omitting the variable as a covariate in the multivariable model. Finally, the baseline platelet count and plasma transfusion volume within 4 days postoperatively were selected as potential confounders and were adjusted in the multivariable logistic regression models. Collinearity analysis showed that there was no collinearity between these covariates and postoperative nadir platelet count.

**Table 2:** Univariable logistic analysis of the risk factors for postoperative cardiovascular complications

Variable	Odds ratio (95% confidence interval)	*P*
Age (years)	1.01 (0.97–1.04)	0.756
Female	1.19 (0.47–3.03)	0.716
Body mass index (kg/m^2^)	0.97 (0.86–1.1)	0.635
Family history of HCM	2.5 (0.75–8.36)	0.137
Family history of sudden death	1.78 (0.42–7.49)	0.433
History of syncope	0.74 (0.26–2.08)	0.571
Duration of clinical symptoms (years)	0.99 (0.91–1.07)	0.757
*Comorbidities*		
Hypertension	1.31 (0.52–3.29)	0.562
Coronary artery disease	1.51 (0.48–4.73)	0.481
Pulmonary arterial hypertension	1.54 (0.37–6.32)	0.551
Diabetes	0 (0–Inf)	0.991
Stoke	1.32 (0.13–13.29)	0.815
Atrial fibrillation	2.76 (0.43–17.59)	0.282
*Preoperative medications*		
Aspirin or Clopidogrel	1.51 (0.43–5.27)	0.517
Diuretics	4.35 (0.82–23.17)	0.085
β- blocker	0.97 (0.37–2.53)	0.945
Statin	1.98 (0.17–22.81)	0.585
ACEI or ARB	0.9 (0.27–3)	0.869
*Laboratory variables*		
Baseline partial thromboplastin time (s)	0.97 (0.71–1.32)	0.833
Baseline APTT (s)	0.95 (0.86–1.04)	0.256
Baseline INR	0.85 (0.25–2.89)	0.799
Baseline hemoglobin (g/L)	1.01 (0.99–1.04)	0.258
Baseline platelet count (10^9^/L)	1 (0.99–1.01)	0.835
*Preoperative echocardiography*		
Left atrium dimension (mm)	1.01 (0.94–1.09)	0.694
Ejection fraction (%)	0.98 (0.91–1.05)	0.549
Maximal septal thickness (mm)	0.99 (0.91–1.07)	0.720
Resting LVOT gradient (mm Hg)	1 (0.99–1.01)	0.870
With systolic anterior motion	1.03 (0.31–3.43)	0.965
*Intraoperative parameters*		
Anesthesia time (min)	1 (1–1.01)	0.125
Surgical time (min)	1.01 (1–1.01)	0.105
Cardiopulmonary bypass time (min)	1.01 (1–1.01)	0.122
Cross-clamp time (min)	1 (0.99–1.02)	0.833
Concomitant other procedure	1.18 (0.45–3.11)	0.736
Hypotension time (min)	1.01 (1–1.01)	0.024
*Intraoperative fluid management*		
Fluid intake (mL)	1 (1–1)	0.407
Red blood cells (units)	0.9 (0.70–1.16)	0.417
Plasma (mL)	1 (1–1)	0.420
Total output (mL)	1 (1–1)	0.460
Blood loss (mL)	1 (1–1)	0.795
Urine volume (mL)	1 (1–1)	0.609
*Blood products within the first 4 days postoperatively*		
Red blood cells (units)	1.4 (1.15–1.71)	0.001
Plasma (mL)	1 (1–1)	< 0.001
Cryoprecipitate	2.16 (0.59–7.92)	0.246
Platelet	6.6 (1.03–42.14)	0.046
Blood loss within first 4 days postoperatively (mL)	1 (1–1)	0.019

Abbreviations: *OR* odds ratio, *CI* confidence interval, *HCM* hypertrophic cardiomyopathy, *ACEI* angiotensin-converting enzyme inhibitor, *ARB* angiotensin receptor blocker, *APTT* activated partial thromboplastin time, *INR* international normalized ratio, *LVOT* left ventricular outflow tract

### Relationship between postoperative nadir platelet count and the risk of cardiovascular complications

The relationship between the postoperative nadir platelet count and the risk of cardiovascular events was assessed using logistic regression analyses (Table 3). Model 1 was unadjusted; model 2 was adjusted for baseline platelet count; and model 3 was additionally adjusted for plasma transfusion volume during the 4 days postoperatively. As a continuous variable, an elevated postoperative nadir platelet count was associated with reduced 30-day postoperative cardiovascular complications. The odds ratios between postoperative nadir platelet count and postoperative cardiovascular events and their confidence intervals are shown in Table 3.

**Table 3:** Multivariable analysis of the association of postoperative nadir platelet count with postoperative cardiovascular complications risk

Models	Odds ratio (95% confidence interval)	*P* value
Model 1	0.98 (0.96–0.99)	0.002
Model 2	0.97 (0.95–0.99)	0.001
Model 3	0.97 (0.95–0.99)	0.005

Model 1 was unadjusted. Model 2 was adjusted for preoperative platelet count. Model 3 was adjusted for preoperative platelet count and plasma transfusion volume during the first 4 postoperative days

**Fig. 2:**
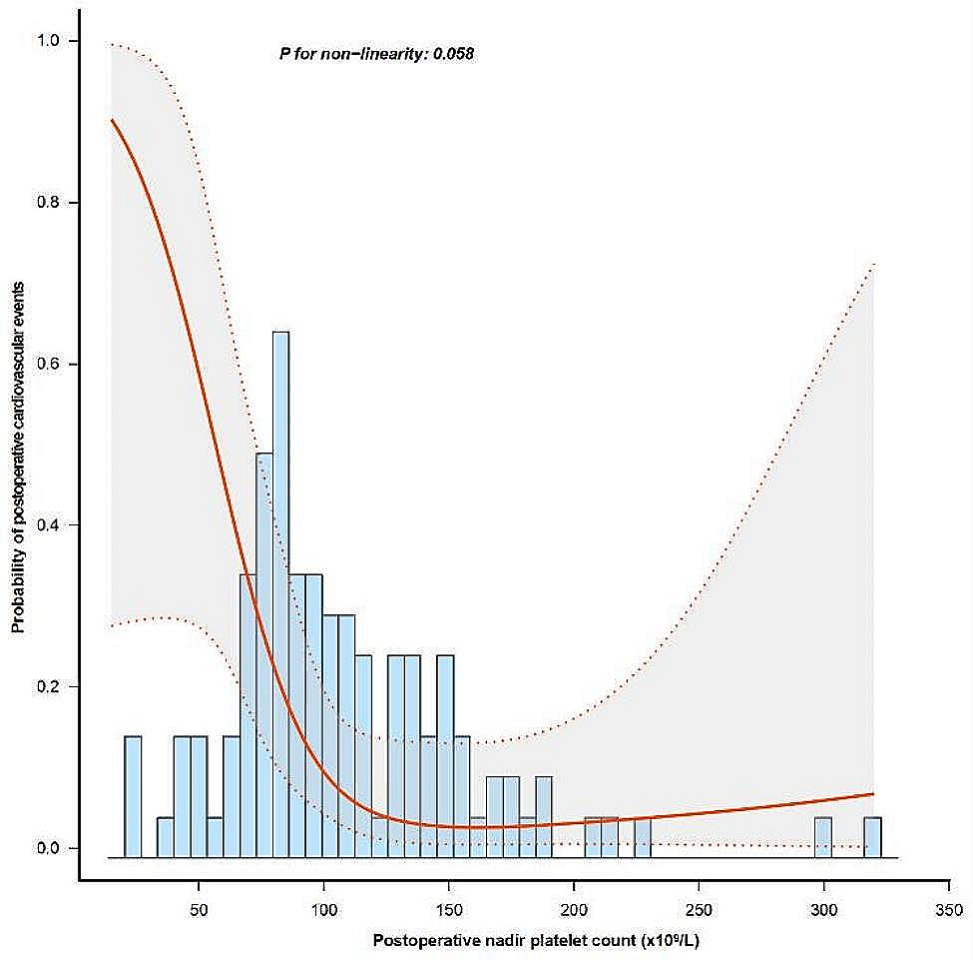
The relationship between postoperative nadir platelet count and postoperative cardiovascular complication risk. The model was adjusted for preoperative platelet count and plasma transfusion within the first 4 days postoperatively. The solid and dashed lines represent the estimated values and 95% confidence intervals

The restricted cubic spline showed that the nadir platelet count was negatively associated with 30-day postoperative cardiovascular complications after full adjustment (Fig. 2, *P*_nonlinearity_ = 0.058).

### Subgroup analysis

**Fig. 3:**
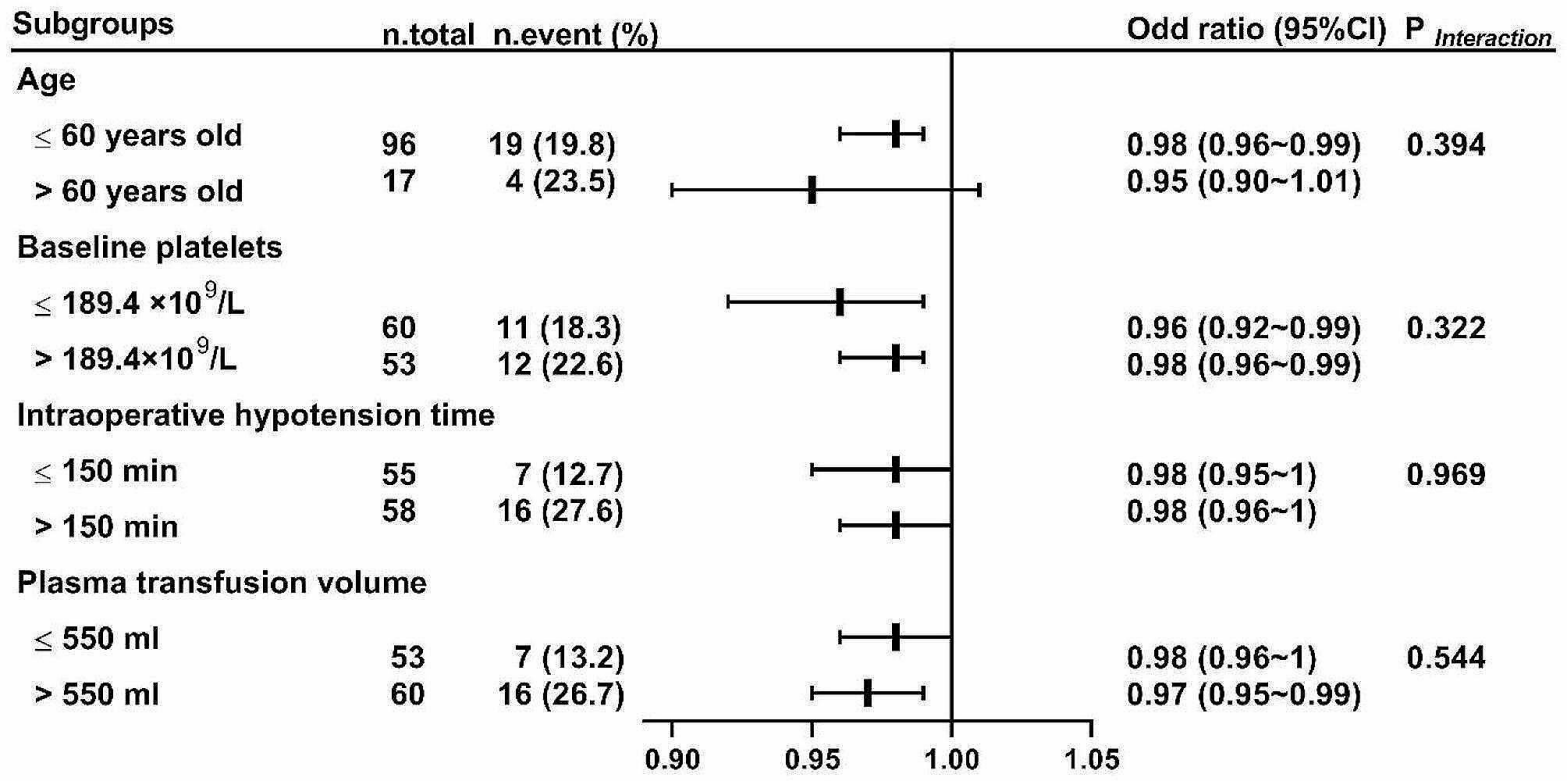
Subgroup analysis of the primary outcome. *OR* odds ratio, *CI* confidence interval

Subgroup analysis details are shown in Fig. 3. The association between nadir platelet count and cardiovascular complications within 30 days post-surgery was consistent in all predefined subgroups (*P*_interaction_ > 0.05).

## Discussion

This retrospective cohort study revealed that the lower postoperative nadir platelet count after septal myectomy was independently associated with increased 30-day postoperative cardiovascular complications in patients with HOCM who underwent septal myectomy and the relationship was linear.

It has been reported that approximately 35–65% of patients undergoing cardiac surgery experience thrombocytopenia, typically during the first 3–4 days post-surgery [18, 23]. The average decrease in platelet count from baseline to immediately after cardiac surgery is 45.0–52.7% [18, 24]. 92% of our patients had a postoperative platelet count decrease (median 42.6% [interquartile range, 29.0–52.4%]), consistent with previous findings. The physiological basis for a decreased platelet count post-cardiac surgery is multifactorial. Hemodilution, blood loss, platelet destruction and activation, and drug-induced thrombocytopenia may be involved [25].

Several clinical studies have indicated that a lower baseline platelet count and platelet activation may be linked to increased cardiovascular complication rates [9–11]. For example, a lower baseline platelet count was associated with a reduced ejection fraction and elevated risk of worsening heart failure among non-surgical patients [9, 10]. A decrease in baseline platelet count is related to myocardial reinfarction risk for patients with ST-elevation myocardial infarction [11]. Cardiopulmonary bypass reduces platelet counts and function [24]. Thus, a decreased postoperative platelet count post-cardiopulmonary bypass may also be related to cardiovascular complications in cardiac surgery patients undergoing cardiopulmonary bypass. However, this potential relationship has not been well-studied to date. Our study showed that postoperative nadir platelet count was significantly associated with the 30-day post-myectomy risk of cardiovascular complications in HOCM patients.

The mechanism underlying the relationship between the postoperative nadir platelet count and cardiovascular complications has not yet been conclusively identified. Previous studies have suggested that platelet destruction, increased platelet reactivity, and microthrombotic state during cardiopulmonary bypass may play essential roles [26]. A transition zone between hemorrhagic and thromboembolic coagulopathy exists. These can occur concurrently in patients post-cardiac surgery [27]. The acquired hemostatic defects induced by platelet destruction during cardiopulmonary bypass can increase the risk of excessive blood loss, re-exploration, and blood transfusion, which are all associated with postoperative morbidity and mortality [27]. Microthrombosis may result in reduced microvascular flow in organs, such as the heart, kidney, and brain, which manifests clinically as myocardial infarction, acute kidney injury, and stroke, and ischemia–reperfusion injury during the postoperative period may aggravate this damage [8, 26]. Moreover, deregulation and activation of platelets after cardiac surgery release numerous inflammatory mediators, thus impairing the body’s ability to resist infection [15].

As this study was conducted retrospectively, it was not possible to establish a causal link between lower postoperative nadir platelet count and higher cardiovascular event rates. However, our study draws attention to the value of postoperative nadir platelet count in identifying patients at increased risk of cardiovascular complications. Routine postoperative platelet count monitoring is a relatively convenient and economical method that is suitable for risk assessment in clinical practice. Future pragmatic research is needed to determine whether serial platelet count measurements during the postoperative period can refine the identification of cardiovascular risk after septal myectomy. Additionally, real-time monitoring of platelet function during the perioperative period, such as using thromboelastography or rotational thromboelastometry, may help guide blood transfusion and may facilitate understanding of the role of platelet count in postoperative cardiovascular complications [28].

This study had some limitations. First, this was a single-center retrospective study. Nevertheless, the homogeneity of a single-center population helps rule out multi-center variations. Additionally, the postoperative thrombocytopenia and cardiovascular complication incidences observed in this study were comparable to those reported in previous large sample-size studies [3, 18]. Second, our sample size was relatively small. However, the present study reported that the primary outcome occurred in 7.1% and 33.3% of patients with higher and lower nadir platelet counts, respectively, with a relative risk of 4.67. Thus, the final sample size of 113 patients provided approximately 90% power to detect the difference with a two-sided level of 0.05. Furthermore, our hospital has offered percutaneous intramyocardial septal radiofrequency ablation, a novel minimally invasive treatment, since 2016. This is becoming an increasingly popular treatment for HOCM patients with drug-refractory symptoms [29], which may limit the number of patients undergoing septal myectomy. Third, compared with previous studies, relatively little platelet transfusion was used in this study, perhaps owing to different transfusion practices at various centers and China’s shortage of concentrated platelets [30]. Fourth, undocumented clinical risk factors may exist. However, we attempted to collect all potential confounding variables that may have affected the study results. Besides, only short-term follow-up data was collected in this study. Larger sample size and prospective clinical trials with mid- or long-term follow-up data are needed in the future. Furthermore, subgroup analyses showed no interaction for subgroups, which may be because of the small sample size. Therefore, the subgroup analysis should be interpreted as exploratory in nature.

## Conclusions

This retrospective cohort study suggested that a lower postoperative nadir platelet count was related to an increased risk of 30-day postoperative cardiovascular complications among adult patients who underwent septal myectomy. Further study is required to determine whether serial platelet measurements during the postoperative period can refine the identification of cardiovascular complication risk in patients undergoing septal myectomy.

## Data Availability

All data can be obtained from the corresponding author upon reasonable request.

## Abbreviations

HOCM: Hypertrophic obstructive cardiomyopathy
LVOT: Left ventricular outflow tract
EHRs: electronic healthcare records
